# Grey matter changes in Meige syndrome: a voxel-based morphology analysis

**DOI:** 10.1038/s41598-020-71479-9

**Published:** 2020-09-03

**Authors:** Jiayu Liu, Lei Li, Lei Chen, Ruen Liu, Yongan Jiang, Jixia Fang, Dongliang Wang, Zhi Liu, Jia Ouyang

**Affiliations:** 1 Department of Neurosurgery, Peking University People’s Hospital, 11th Xizhimen South St., Beijing, 100044 China; 2 Department of Nuclear Medicine, Zhuhai People’s Hospital (Zhuhai hospital affiliated with Jinan University), No. 79 Kangning Road, Xiangzhou District, Zhuhai, 519000 Guangdong China; 3 Department of Radiology, Peking University People’s Hospital, 11th Xizhimen South St., Beijing, 100044 China; 4 Department of Neurosurgery Jiangxi Provincial People’s Hospital Affiliated to Nanchang University, Nanchang, 330006 Jiangxi China

**Keywords:** Neuroscience, Neurology

## Abstract

To investigate the changes and clinical significance of brain structural abnormalities in patients with Meige syndrome and related depressive symptoms. We retrospectively analysed clinical data, imaging examinations, and Hamilton Depression Rating scale scores in 46 patients with Meige syndrome from January 2017 to January 2019. We compared the Meige syndrome group with the healthy control group, and the definite depression group with the non-definite depression group. Voxel-based morphometry (VBM) was used to compare grey matter (GM) volumes. We conducted two-sample t-tests corrected for subject age and gender. We tested at a level of significance of p < 0.001 with a false discovery rate (FDR) correction. VBM demonstrated decreased GM volume (p < 0.001 and cluster size > 50 voxels) in the left hemisphere in the middle frontal orbital gyrus, temporal pole (superior temporal gyrus) and insula and in the right hemisphere in the temporal pole (middle temporal gyrus), precuneus, inferior parietal, inferior temporal and olfactory cortices in the Meige syndrome group. Comparing VBM-MRI measures in Meige syndrome patients with and without depression, decreased GM volume was found in the left hemisphere in the cuneus and hippocampus and in the right hemisphere in the angular gyrus, middle frontal gyrus and middle occipital gyrus in the definite depression group. Unlike other dystonia studies that have suggested an involvement of the basal ganglia and motor cortex in the pathophysiology of the disorder , we believe that the precuneus is involved in the development of Meige syndrome. Additionally, our findings suggest that the hippocampus plays a role in the pathogenesis of depression in patients with Meige syndrome.

## Introduction

Meige syndrome, also known as idiopathic blepharospasm-oromandibular dystonia syndrome, is a rare type of segmental dystonia disorder with features of adult hyperactivity disorder and characterized by blepharospasm and oromandibular dystonia disorder^1–3^. Meige syndrome usually occurs between 30 and 60 years of age, with rare cases appearing in teenage years^4^. This disease is more common in females, and the ratio of males to females is approximately 1:3. Meige syndrome can be divided into three types according to clinical manifestations: blepharospasm, blepharospasm-oromandibular dystonia, and oromandibular dystonia, among which blepharospasm with oromandibular dystonia is regarded as the complete type of Meige syndrome^3^. Meige syndrome is classified into primary and secondary Meige syndromes. Although plastic changes and reduced cortical inhibition have been documented by neurophysiological and neuroimaging techniques^5^, the aetiology of primary Meige syndrome is still unknown.

In recent years, the application of the voxel-based morphometry method of magnetic resonance imaging (VBM-MRI) for structural imaging research has provided a new and important method for structural research of neurologic movement disorders^6^. Some VBM-MRI studies have shown significant reductions in grey matter (GM) volume mainly in the cerebellar vermis and bilateral superior frontal gyri^7,8^. However, they did not study Meige patients, and the number of included patients was small. Therefore, a VBM study on Meige syndrome is necessary to clarify the pathogenesis of this disease. In addition to blepharospasm and oromandibular dystonia, Meige syndrome is more commonly associated with mood disorders, with a higher incidence of depression^3^. In this study, VBM analysis was used to investigate the changes and clinical significance of brain structural abnormalities in patients with Meige syndrome and related depressive symptoms.

## Results

### Baseline characteristics

A total of 46 cases (11 males; 35 females) were included, and the age of the participants ranged from 37 to 73 years (57.00 ± 8.86). The duration of Meige syndrome ranged from 3 to 8 years (4.57 ± 2.23). Blepharospasm was present in 18 patients, and 5 had oromandibular dystonia; 23 patients had both blepharospasm and oromandibular dystonia. The BFMDRS movement scores of participants ranged from 3.25 to 13.5 (7.87 ± 2.45). The HAMD scores of the participants ranged from 9 to 29 (16.96 ± 4.84). In the control group, a total of 64 individuals (25 males; 39 females) were included, and the average age was 52.71 ± 6.26. The BFMDRS and HAMD scores were significantly higher in the Meige syndrome group than in the healthy control group (P < 0.05) (Table 1).

**Table 1:** Clinical data for Meige syndrome patients and control subjects.

	Patients	Controls	P value
Gender (female)	35	39	0.104
Age (years)	57.00 ± 8.86	52.71 ± 6.26	0.083
BFMDRS movement scores	7.87 ± 2.45	0.43 ± 0.49	0.000
HAMD scores	16.96 ± 4.84	5.51 ± 2.95	0.000
Disease duration (years)	4.57 ± 2.23	–	–
Distribution (n)		–	–
Blepharospasm	18	–	–
Oromandibular dystonia	5	–	–
Both	23	–	–
Total	46	64	–

We divided the patients into a definite depression group (< 18) and a non-definitive depression group (> 18) according to HAMD scores. Finally, there were 25 patients in the definitive depression group and 21 in the non-definitive depression group. There were no significant differences between the two groups in terms of age, gender or BFMDRS movement scores (P > 0.05) (Table 2).

**Table 2:** Clinical data for Meige syndrome patients with and without depression.

	Depression group	Non-depression group	P value
Gender (female)	18	17	0.514
Age (years)	56.96 ± 9.91	57.67 ± 7.33	0.111
BFMDRS movement scores	9.56 ± 1.62	6.17 ± 1.38	0.726

### Comparison of VBM-MRI in patients with Meige syndrome and controls

**Figure 1:**
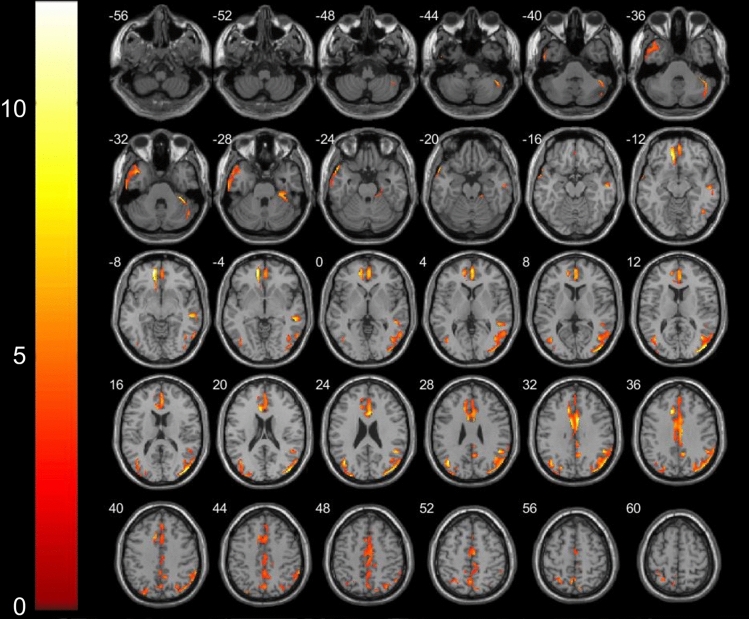
Areas showing decreased GM volume in axial slices (p < 0.001) in Meige syndrome patients and control subjects. In the left hemisphere in the middle frontal orbital gyrus (t = − 8.83, p < 0.001), temporal pole superior temporal gyrus (t = − 8.20, p < 0.001) and insula (t = − 7.98, p < 0.001); in the right hemisphere in the temporal pole middle temporal gyrus (t = − 7.32, p < 0.001), precuneus (t = − 5.99, p < 0.001), inferior parietal (t = − 5.84, p < 0.001), inferior temporal (t = − 5.03, p < 0.001)and olfactory cortices (t = − 4.95, p < 0.001).

Conventional MRI showed normal results in all patients with Meige syndrome and controls. The VBM analysis in the current study revealed multiple significant differences between the patients with Meige syndrome and controls. We found decreased GM volume (p < 0.001 and cluster size > 50 voxels) in the left hemisphere in the middle frontal orbital gyrus, temporal pole (superior temporal gyrus) and insula and in the right hemisphere in the temporal pole (middle temporal gyrus), precuneus, inferior parietal, inferior temporal and olfactory cortices (Table 3; Fig. 1).

**Table 3:** Areas of decreased GM volume (p < 0.001) in Meige syndrome patients and control subjects.

Structure	Number of voxels	Peak MNI coordinate			P value	T value
		x	y	z		
Frontal mid Orb_L	424	− 19.5	63	− 15	< 0.001	− 8.83
Temporal pole Sup_L	1,231	− 54	15	− 22.5	< 0.001	− 8.20
Insula_L	107	− 40.5	15	− 6	< 0.001	− 7.98
Temporal pole Mid_R	260	42	10.5	− 31.5	< 0.001	− 7.32
Precuneus_R	243	12	− 55.5	67.5	< 0.001	− 5.99
Parietal Inf_R	86	37.5	− 55.5	45	< 0.001	− 5.84
Temporal_Inf_R	196	58.5	− 12	− 36	< 0.001	− 5.03
Olfactory_R	71	6	22.5	− 4.5	< 0.001	− 4.95

### Comparison of VBM-MRI in patients with Meige syndrome with and without depression

**Figure 2:**
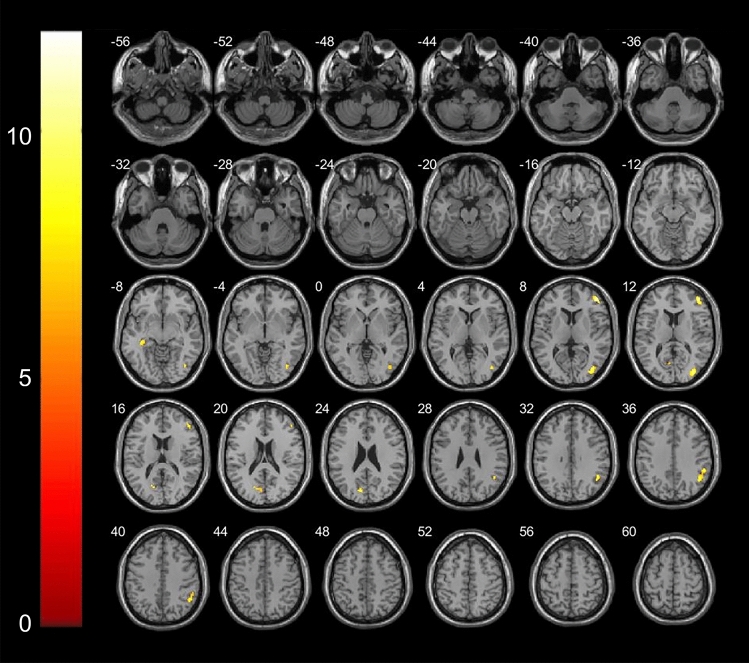
Areas showing decreased GM volume in axial slices in Meige syndrome patients with and without depression. In the left hemisphere in the cuneus (t = − 4.82, p < 0.001) and hippocampus (t = − 4.23, p < 0.001); in the right hemisphere in the angular gyrus(t = − 4.84, p < 0.001), middle frontal gyrus(t = − 6.29, p < 0.001) and middle occipital gyrus(t = − 5.80, p < 0.001).

We found decreased GM volume (p < 0.001 and cluster size > 50 voxels) in the left hemisphere in the cuneus and hippocampus and in the right hemisphere in the angular gyrus, middle frontal gyrus and middle occipital gyrus (Table 4; Fig. 2).

**Table 4:** Areas of decreased GM volume (p < 0.001) in Meige syndrome patients with and without depression.

Structure	Number of voxels	Peak MNI coordinate			P value	T value
		x	y	z		
Angular_R	203	46.5	− 54	34.5	< 0.001	− 4.84
Cuneus_L	159	− 10.5	− 76	22.5	< 0.001	− 4.82
Frontal mid_R	284	45	45	9	< 0.001	− 6.29
Hippocampu_L	52	− 34.5	− 30	− 7.5	< 0.001	− 4.23
Occipital mid_R	51	36	− 82.6	10.5	< 0.001	− 5.80

## Discussion

Meige syndrome was named after French neurologist Henry Meige. In 1910, Henry Meige described approximately 10 patients with blepharospasm, one of whom also had mandibular dystonia^9^. Currently, most researchers and clinicians prefer the term “Meige syndrome” to describe blepharospasm-oromandibular dystonia syndrome^10^. Meige syndrome usually occurs between 30 and 60 years of age, with rare cases presenting during teenage years^4^. This disease is more common in females, and the ratio of males to females is approximately 1:3^11^. In our study, a total of 46 patients (11 males; 35 females) were included, and the age of the participants ranged from 37 to 73 years (57.00 ± 8.86), which was consistent with previous studies. Meige syndrome can be divided into three types according to clinical manifestations: blepharospasm, blepharospasm-oromandibular dystonia, and oromandibular dystonia, among which blepharospasm with oromandibular dystonia is regarded as the complete type of Meige syndrome^10^. In our study, blepharospasm was present in 18 patients, and 5 had oromandibular dystonia; 23 patients had both blepharospasm and oromandibular dystonia. The BFMDRS movement scores of participants ranged from 3.25 to 13.5 (7.87 ± 2.45).

## Discussion

The aetiology of primary Meige syndrome is still unknown, but changes in plasticity and reduced cortical inhibition due to environmental triggers and genetic susceptibility may be associated with the onset of Meige syndrome^5^. Many movement disorders are not limited to a particular region but involve multiple brain regions^12^. Therefore, morphological studies of the whole brain are helpful to understand the pathogenesis of Meige syndrome. VBM techniques quantitatively analyses the GM and WM volumes in each voxel in MRI images, thereby allowing an evaluation of the anatomical structural differences across the whole brain^13^. Recently, researchers using VBM observed conflicting results of increased, decreased, or no change in GM volume in patients with primary blepharospasm and cervical dystonia^8^. A VBM study by Piccinin^7^ showed that patients with craniocervical dystonia had reduced GM volume in the cerebellar vermis, bilateral superior frontal gyri, precuneus, cingulate, insular cortex, and corpus callosum fissure. However, all of these studies examined patients with craniocervical dystonia, and to the best of our knowledge, there is no research on Meige syndrome.

In this study, VBM analysis was used to investigate the changes and clinical significance of brain structural abnormalities in patients with Meige syndrome. We found decreased GM volume (p < 0.001 and cluster size > 50 voxels) in the left hemisphere in the middle frontal orbital gyrus, temporal pole (superior temporal gyrus) and insula and in the right hemisphere in the temporal pole (middle temporal gyrus), precuneus, inferior parietal, inferior temporal and olfactory cortices. We failed to detect any abnormalities in the basal ganglia and motor cortex. The results from previous VBM-MRI-related studies in those with dystonia have shown varying increases and decreases in GM volume in the basal ganglia^14,15^, and no abnormalities have been found in the basal ganglia in recent years^16^, which is consistent with the results of this study. However, a recent study showed that the precentral gyrus, which plays an important role in motor planning and learning, is involved in the onset of craniocervical dystonia^7^. One of the reasons why our results differ from the above results is that the patients they selected were not typical Meige syndrome patients^7,14,16^. The present study is the first VBM-MRI study in patients with Meige syndrome, and the sample size of this study was larger than previous studies. Therefore, although functional changes in brain regions such as the basal ganglia are necessary for the development and maintenance of dystonia, they do not necessarily result in anatomical changes^7^.

In this study, we found a reduction in GM volume in the precuneus and inferior parietal cortex in patients with Meige syndrome. The precuneus lies deep in the parietal lobe and interacts with the adjacent posterior medial cortex (posterior cingulate and posterior compressed cortex). This internal connection is bilateral, providing communication between corresponding components in the two hemispheres and to some extent providing the anatomical basis for bilateral functional coordination^17^. The precuneus is also selectively associated with other parietal regions, such as the supraparietal lobule, and is involved in visual spatial information processing. Recent studies have found that the precuneus is associated with many higher cognitive functions, such as episodic memory, self-related information processing, and consciousness, and is no longer considered a simple extension of the lateral parietal cortex^18^. Therefore, abnormal findings in the precuneus also indicated that patients with Meige syndrome may have abnormal integration at the cortical level. The precuneus has been shown to have extensive connections with the auxiliary motor areas. At the subcortical level, the modular prefrontal lobe is primarily associated with the nuclei of the basal ganglia and projects fibres into the brainstem^19^. Therefore, we hypothesized that the precuneus plays an important role in the pathogenesis of Meige syndrome, which partly explains the lack of GM volume reductions in the basal ganglia and motor cortex.

In the frontal lobe, we found a reduction in GM volume in the left middle frontal orbital gyrus in the patients with Meige syndrome. The frontal lobe plays a key role in many of the brain’s higher functions, including attention regulation, learning and memory, behaviour planning, behavioural inhibition, thinking and reasoning^20^. At the same time, the frontal lobe is also an important part of “emotional centre” or “emotion-related pathways”^21^. The orbitofrontal cortex is involved in the encoding of external and internal information, reward-related behaviour, impulse control, and emotional regulation^22^. The prefrontal cortex is also extensively connected to the superior temporal gyrus and olfactory cortex^22^. In the temporal lobe, we also found that the patients with Meige syndrome had decreased GM volume in the superior temporal pole, middle temporal pole and inferior temporal regions. At present, it is believed that the temporal lobe plays an important role in visual and auditory integration and regulation^23^ and is closely related to face recognition, distance judgement and text comprehension in reading. Many Meige syndrome patients have varying degrees of depression and other emotional disorders. Therefore, we believe that the decrease in GM volume in the frontal lobe and temporal lobe in this study was mainly related to the non-motor symptoms of this disease. Finally, we found that the patients with Meige syndrome had decreased GM volume in the insula. The posterior insular cortex is mainly responsible for the integration of sensorimotor functions in the brain together with the primary motor cortex, sensorimotor cortex, auxiliary motor area and middle cingulate gyrus, while the anterior insular cortex is mainly responsible for emotional regulation together with the anterior cingulate gyrus, middle temporal gyrus and inferior temporal gyrus^24,25^. Therefore, we believe that the insula may be involved in the development of motor and non-motor symptoms of Meige syndrome.

Meige syndrome with mood disorders is also common, with a higher incidence of depression than in the general population^3^. In our study, the severity of depression was determined by the psychological 21-item Hamilton Depression Rating scale (HAMD)^26^. There were 25 patients in the definite depression group. When we compared VBM-MRI measures in Meige syndrome patients with and without depression, we found decreased GM volume (p < 0.001 and cluster size > 50 voxels) in the left hemisphere in the cuneus and hippocampus and in the right hemisphere in the angular gyrus, middle frontal gyrus and middle occipital gyrus (Table 4; Fig. 2). The results of this study suggest that, in addition to the frontal lobes mentioned above, the hippocampus plays an important role in the pathogenesis of depression in patients with Meige syndrome. A previous study has shown not only that depression is manifested by bilateral hippocampal volume reduction but also that the degree of reduction is related to the severity of symptoms, the course of disease and the number of recurrences^27^. The patients in that study had a long duration of illness (4.57 ± 2.23 years), and the hippocampal changes accompanying depression also showed a process of gradual accumulation, which finally manifested as volume reduction, which was consistent with the results of the present study. The occipital lobe is involved in attention, visual memory, visual movement, language movement and other neuropsychological activities^28^. In the process of visual information processing of external positive stimuli in the basic state of chronic or recurrent depression, the function of the relevant cortex was weaker than that in healthy controls. The cuneus is also part of the occipital lobe, and we believe that it is involved in the development of depression in patients with Meige syndrome.

## Conclusion

At present, there are few imaging studies on the pathogenesis of Meige syndrome, and most of them include a heterogeneous collection of patients with dystonias. In this present study, VBM analysis was used to investigate the changes and clinical significance of brain structural abnormalities in patients with Meige syndrome and related depressive symptoms. First, unlike previous imaging studies with patients with dystonia, we found no abnormalities in the basal ganglia and motor cortex. However, the important role of the precuneus in the pathogenesis of Meige syndrome may have been revealed in our study. Second, the decrease in GM volume in the frontal lobe and temporal lobe in this study was mainly related to the non-motor symptoms of this disease. Finally, we compared VBM-MRI in Meige syndrome patients with and without depression and found that the hippocampus plays an important role in the pathogenesis of depression in patients with Meige syndrome.

The limitation of this study is that it is only a cross-sectional study with a relatively small sample size. The sample size can be further expanded in the future. In addition, positron emission tomography (PET) is an in vivo method that reflects the level of brain metabolism. Combined with VBM-MRI, a better understanding of the changes in brain function and morphology in Meige syndrome can be obtained. Another limitation of our study is that the cohort is composed of a mix of significantly different phenotypes of Meige syndrome (oromandibular, blepharospasm and combined oromandibular and blepharospasm). In the future, our team plans to investigate into the similarities and differences in gray matter volume across these disorders.

## Methods

### Patients

Clinical data of 46 right-handed patients with primary Meige syndrome were collected between January 2017 and January 2019 at the Department of Neurosurgery, Peking University People’s Hospital. Primary Meige syndrome diagnosed by an experienced neurologist, Ruen Liu. The diagnostic criteria are mainly based on blepharospasm, oral and mandibular dystonia, increased blink rates and other symptoms. Laboratory and imaging examinations showed no abnormalities. Patients with histories of drug or alcohol use disorders, psychiatric disorders and other secondary factors were excluded. Based on the clinical manifestations, the patients were divided into three types: (1) blepharospasm; (2) blepharospasm with oromandibular dystonia; and (3) oromandibular dystonia. In this study, 64 right-handed healthy people with age and education levels matching the Meige syndrome group were included as the healthy control group.

Written informed consent was obtained from each participant, and the study was approved by the institutional review board of Peking University People’s Hospital. All methods were carried out in accordance with relevant guidelines and regulations.

### Magnetic resonance imaging

Imaging was conducted on a Discovery 750 3.0 T (GE Healthcare, Waukesha, WI) MRI scanner. T1-weighted anatomical images in the sagittal plane were collected with a 3D fast spoiled gradient echo sequence: repetition time (TR) = 4.9 ms, echo time (TE) = 2 ms, flip angle = 15°, field of view (FOV) = 240 mm, in-plane resolution = 1 × 1 mm^2^, slice thickness = 1 mm, and 170 slices. All scans were performed by the same imaging physician.

### Image data analysis

Structural images were processed with SPM8 (https://www.fil.ion.ucl.ac.uk/spm) and VBM8 toolbox (https://dbm.neuro.unijena.de/vbm) within the MATLAB R2016a programming environment (The MathWorks, Natick, MA).

Preprocessing of the data involved spatial normalization, GM segmentation and spatial smoothing with a Gaussian kernel. Image preprocessing mainly included the following steps: (1) with the aid of the MATLAB platform, SPM8 converted the collected images to a format that can be recognized and uses MRIcro software (https://www.mccauslandcenter.sc.edu/mricro/mricro/mricro.html) to adjust the spatial coordinates of each image such that the data from all subjects had the spatial coordinates of the image adjusted and all participants' images were located in a closed spatial coordinate system. Since the brain structure of each subject greatly varied, we needed to conduct spatial standardization on all the images from the subjects and convert them into standardized images with the same size and direction; (2) according to the distribution template of GM, white matter (WM) and cerebrospinal fluid (CSF) in the brain, each element in the image was judged to be GM, WM or CSF to segment the GM, WM or CSF and generate different brain tissue images^29^; (3) the last step is Gaussian smoothing of the standardized image. The image obtained in the previous step was smoothed by a Gaussian kernel of 8 mm × 8 mm × 8 mm at half height and width. This step can eliminate high-frequency noise and improve the signal-to-noise ratio of the image. In addition, the image can be made to have the characteristics of a random Gaussian field and meet the requirements of the statistical analyses of SPM.

### Data collection

Baseline data and medical histories were obtained from patient medical records. Baseline data included age, sex, and dystonia and depressive status. Dystonia was evaluated using the Burke–Fahn–Marsden Dystonia Rating Scale (BFMDRS)^30^. The severity of depression was determined by the psychological 21-item Hamilton Depression Rating scale (HAMD)^26^. A score of less than 7 was considered normal; 7–17 indicated possible depression; 18 to 24 indicated definite depression; and greater than 24 was considered severe depression. Finally, we divided the patients into a definite depression group (< 18) and a non-definite depression group (> 18).

### Statistical analysis

SPSS 19.0 statistical software (IBM Corp., Armonk, NY, USA) was used for data analysis^31^. Numerical variables are expressed as the mean ± standard deviation. Qualitative variables are described by absolute values of cases in different groups. Statistical significance between the quantitative variables was assessed by X2 tests, corrected by Yates or Fisher if necessary. Student’s t-tests were performed to evaluate data that followed a normal distribution. P < 0.05 was considered statistically significant differences.

Two-sample t-tests corrected for subject age, gender and total intracranial volume were used for VBM-MRI comparisons between groups^7^. Significant differences between groups were indicated when P < 0.001 (false discovery rate corrected). Only sets with more than 50 voxels were considered statistically significant brain regions. The t-test results are superimposed on the template of the T1 structural image, and the results are presented by the xjView software (version 9.6, https://www.alivelearn.net/xjView).

### Ethics approval

The present study was approved by the Medical Ethics Committee of Peking University People’s Hospital. All methods were carried out in accordance with relevant guidelines and regulations.

### Consent to participate

All patients provided written informed consent to participate.

### Consent for publication

The study participants provided their consent for the publication of any data/associated images.

## Data Availability

The datasets used and/or analysed during the current study are available from the corresponding author upon reasonable request.

## References

[1] Tolosa ES (1981). Clinical features of Meige's disease (idiopathic orofacial dystonia): a report of 17 cases. Arch. Neurol..

[2] Miao J (2010). Meige's syndrome and hemichorea associated with hyperthyroidism. J. Neurol. Sci..

[3] Jahngir MU, Patel BC (2020). StatPearls.

[4] Sabesan T (2008). Meige syndrome: a rare form of cranial dystonia that was treated successfully with botulinum toxin. Br. J. Oral. Maxillofac. Surg..

[5] Hallett M (2002). Blepharospasm: recent advances. Neurology.

[6] Barrett MJ (2019). Lower volume, more impairment: reduced cholinergic basal forebrain grey matter density is associated with impaired cognition in Parkinson disease. J. Neurol. Neurosurg. Psychiatry.

[7] Piccinin CC (2014). Diffuse decreased gray matter in patients with idiopathic craniocervical dystonia: a voxel-based morphometry study. Front. Neurol..

[8] Valls-Sole J, Defazio G (2016). Blepharospasm: update on epidemiology, clinical aspects, and pathophysiology. Front. Neurol..

[9] Pandey S, Sharma S (2017). Meige's syndrome: history, epidemiology, clinical features, pathogenesis and treatment. J. Neurol. Sci..

[10] LeDoux MS (2009). Meige syndrome: what's in a name?. Parkinsonism Relat. Disord..

[11] Soland VL, Bhatia KP, Marsden CD (1996). Sex prevalence of focal dystonias. J. Neurol. Neurosurg. Psychiatry.

[12] Braak H (2003). Staging of brain pathology related to sporadic Parkinson's disease. Neurobiol. Aging.

[13] Good CD (2001). A voxel-based morphometric study of ageing in 465 normal adult human brains. Neuroimage.

[14] Pantano P (2011). A transverse and longitudinal MR imaging voxel-based morphometry study in patients with primary cervical dystonia. AJNR Am. J. Neuroradiol..

[15] Draganski B, Thun-Hohenstein C, Bogdahn U, Winkler J, May A (2003). "Motor circuit" gray matter changes in idiopathic cervical dystonia. Neurology.

[16] Martino D (2011). Cortical gray matter changes in primary blepharospasm: a voxel-based morphometry study. Mov. Disord..

[17] Seitz RJ, Binkofski F (2003). Modular organization of parietal lobe functions as revealed by functional activation studies. Adv. Neurol..

[18] Vogeley K (2004). Neural correlates of first-person perspective as one constituent of human self-consciousness. J. Cogn. Neurosci..

[19] Leichnetz GR (2001). Connections of the medial posterior parietal cortex (area 7m) in the monkey. Anat. Rec..

[20] Miller EK, Cohen JD (2001). An integrative theory of prefrontal cortex function. Annu. Rev. Neurosci..

[21] Cacioppo JT, Gardner WL (1999). Emotion. Annu. Rev. Psychol..

[22] Price JL, Drevets WC (2012). Neural circuits underlying the pathophysiology of mood disorders. Trends Cogn. Sci..

[23] Ono Y (2014). Frontotemporal oxyhemoglobin dynamics predict performance accuracy of dance simulation gameplay: temporal characteristics of top-down and bottom-up cortical activities. Neuroimage.

[24] Le Berre AP (2017). Deviant functional activation and connectivity of the right insula are associated with lack of awareness of episodic memory impairment in nonamnesic alcoholism. Cortex.

[25] Zhang Y (2017). Abnormal functional connectivity of ventral anterior insula in asthmatic patients with depression. Neural Plast.

[26] Williams JB (1988). A structured interview guide for the Hamilton depression rating scale. Arch. Gen. Psychiatry.

[27] Cole J, Costafreda SG, McGuffin P, Fu CH (2011). Hippocampal atrophy in first episode depression: a meta-analysis of magnetic resonance imaging studies. J. Affect Disord..

[28] Jiang T, He Y, Zang Y, Weng X (2004). Modulation of functional connectivity during the resting state and the motor task. Hum. Brain Mapp..

[29] Ashburner J, Friston K (1997). Multimodal image coregistration and partitioning: a unified framework. Neuroimage.

[30] Burke RE (1985). Validity and reliability of a rating scale for the primary torsion dystonias. Neurology.

[31] Liu J (2020). Clinical analysis of patients with ipsilateral coexistence of hemifacial spasm and trigeminal neuralgia. World Neurosurg..

